# Non-invasive and fully two-dimensional quantitative visualization of transparent flow fields enabled by photonic spin-decoupled metasurfaces

**DOI:** 10.1038/s41377-025-01793-2

**Published:** 2025-03-05

**Authors:** Qingbin Fan, Peicheng Lin, Le Tan, Chunyu Huang, Feng Yan, Yanqing Lu, Ting Xu

**Affiliations:** 1 National Laboratory of Solid-State Microstructures and Collaborative Innovation Center of Advanced Microstructures, Nanjing University, Nanjing, 210093 China; 2 School of Electronic Sciences and Engineering, Nanjing University, Nanjing, 210093 China; 3 College of Engineering and Applied Sciences and Key Laboratory of Intelligent Optical Sensing and Manipulation, Ministry of Education, Nanjing University, Nanjing, 210093 China; 4 School of Materials Engineering, Jiangsu University of Technology, 213001 Changzhou, China

**Keywords:** Metamaterials, Sub-wavelength optics

## Abstract

Transparent flow field visualization techniques play a critical role in engineering and scientific applications. They provide a clear and intuitive means to understand fluid dynamics and its complex phenomena, such as laminar flow, turbulence, and vortices. However, achieving fully two-dimensional quantitative visualization of transparent flow fields under non-invasive conditions remains a significant challenge. Here, we present an approach for achieving flow field visualization by harnessing the synergistic effects of a dielectric metasurface array endowed with photonic spin-decoupled capability. This approach enables the simultaneous acquisition of light-field images containing flow field information in two orthogonal dimensions, which allows for the real-time and quantitative derivation of multiple physical parameters. As a proof-of-concept, we experimentally demonstrate the applicability of the proposed visualization technique to various scenarios, including temperature field mapping, gas leak detection, visualization of various fluid physical phenomena, and 3D morphological reconstruction of transparent phase objects. This technique not only establishes an exceptional platform for advancing research in fluid physics, but also exhibits significant potential for broad applications in industrial design and vision.

## Abstract (web summary)

Non-invasive, fully two-dimensional visualization of transparent flow fields enabled by metasurface.

## Graphical abstract

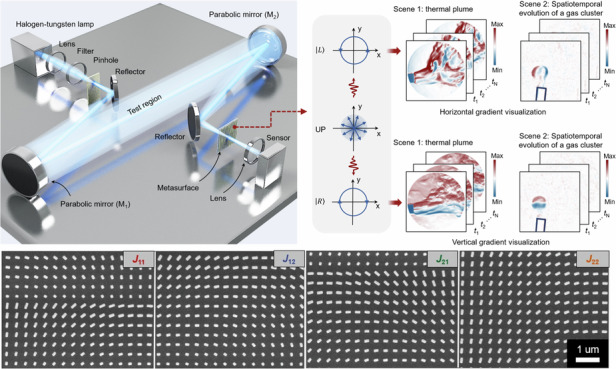

## Introduction

Flow fields, including fluids and gases, are typically transparent and therefore invisible to the naked eye or conventional cameras. Consequently, transparent flow field visualization is a critical technology for numerous scientific and engineering disciplines, such as investigations of shock-wave structures,^1^ combustion chamber flows,^2^ wind tunnels,^3^ and boundary layers.^4^ Visualizing flow fields not only enhances our comprehension of fundamental physical phenomena, but also significantly advances the development of efficient engineering systems.

A wide range of techniques have been developed to acquire information within transparent flow fields. For instance, the probe method provides high temporal resolution for point-specific measurements but introduces disturbances into the flow due to its invasive nature, making it less suitable for specific applications.^5,6^ Particle image velocimetry (PIV) effectively tracks flow patterns and measures velocity fields, particularly in complex flows, but it requires seeding the flow with particles, which may not be feasible in all environments, and it lacks direct access to other flow properties like pressure or temperature.^7–9^ Laser-induced fluorescence (LIF) allows visualization of intricate flow patterns, turbulence structures, and chemical reactions, making it a powerful tool in experimental fluid dynamics.^10,11^ However, introducing fluorescent tracers may slightly alter the natural flow properties, and its applicability is limited to flows where such tracers can be safely and effectively introduced. Conventional schlieren techniques can acquire flow field information through optical methods but provide only one-dimensional information that depends on the direction of the spatial filter.^12^ In contrast, the background-oriented schlieren (BOS) technique has been widely applied across various scenarios.^13^ It enables high-resolution measurements at microscopic scales and is also effective for large-scale observations, including fields extending hundreds of meters and even supersonic aircraft in flight.^14,15^ Despite these advantages, BOS requires specific backgrounds and involves complex post-processing steps. Another technique is infrared thermal imaging, which provides non-invasive detection of temperature gradients, making it valuable for studying thermal effects specifically on flow surfaces. However, its sensitivity is limited to detecting surface radiation, which restricts its ability to characterize temperature distributions throughout the entire flow field.^16–18^ Overall, while existing techniques each offer unique advantages for studying transparent flow fields, they also present significant limitations, including invasiveness, the need for specific tracers, complex post-processing algorithms, or restricted dimensions.

Optical metasurfaces, which consist of arrays of subwavelength-spaced plasmonic or dielectric optical scatters at an ultrathin interface, promote the advancement of optical technology by enabling versatile functionalities in a planar structure.^19–27^ By spatially controlling the metasurface scatter geometrical parameters (such as size, shape, and orientation), one can shape the wavefronts of transmitted or reflected light with high spatial resolution. Meanwhile, the field of metasurface polarization optics has been developed, allowing metasurfaces to possess additional degrees of freedom, thereby enabling the realization of multifunctional devices.^28–32^ Photonic spin-decoupled metasurfaces represent a class of multifunctional devices that can provide distinct functionalities for left-circularly polarized (LCP) and right-circularly polarized (RCP) light. In recent years, metasurfaces have exhibited unprecedented advantages in imaging applications, such as polarization imaging,^33–36^ hyperspectral imaging,^37,38^ light-field imaging,^39,40^ phase gradient microscopes^41,42^ and augmented reality displays.^43,44^ Despite these significant advances, applications of metasurfaces for transparent flow field visualization have yet to be explored.

In this work, we implement non-invasive, fully two-dimensional transparent flow field visualization using the synergistic effects of the dielectric metasurface array endowed with photonic spin-decoupling capability. In transparent flow fields, density gradient vectors in both horizontal (*x*-axis) and vertical directions (*y*-axis) can perturb light propagating along the *z*-axis. Detecting these perturbation signals enables the acquisition of the required flow field information. By using the photonic spin-decoupled metasurface array, we have accomplished tasks previously unachievable with conventional optics, establishing a mapping correlation between polarization states and the density gradient vectors in two orthogonal dimensions. This enables the simultaneous obtainment of light-field images containing flow field information in two orthogonal dimensions, which facilitates the quantitative derivation of multiple physical parameters in a single shot. As a proof-of-concept, we experimentally demonstrate the versatility of the proposed visualization technique across diverse applications, including temperature field mapping, gas leak detection, visualization of various fluid physical phenomena, and 3D morphology reconstruction of transparent phase objects. This approach not only provides a robust platform for advancing fluid physics research but also holds considerable promise for a wide range of industrial design and detection applications.

## Results

### Principle of metasurface-based transparent flow field visualization

**Fig. 1:**
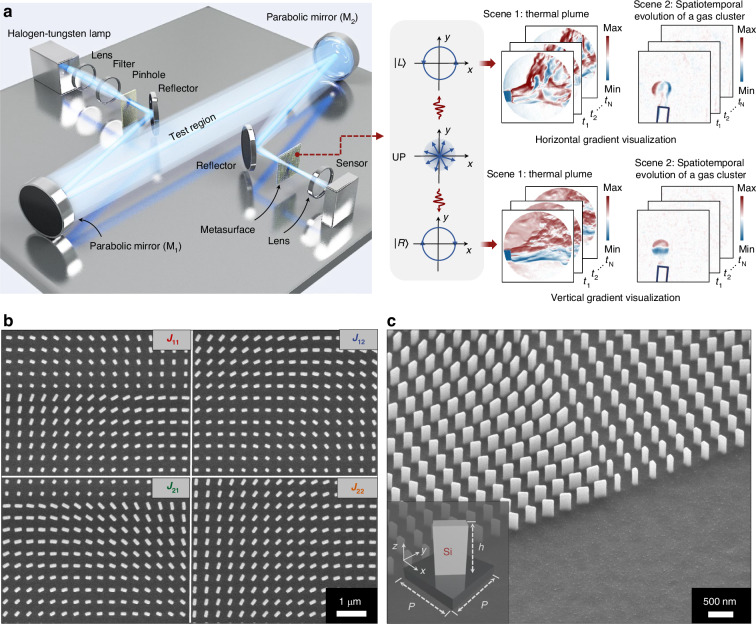
Conceptual illustration of non-invasive, fully two-dimensional visualization of transparent flow fields using photonic spin-decoupled metasurface. **a** Left panel: schematic of the experimental setup. The distance between the first (M_1_) and second (M_2_) parabolic mirrors is ~5 m to ensure a sufficiently large test area. Both parabolic mirrors have the same diameter (15 cm) and focal length (1.5 m). The metasurface is positioned at the focal plane of the parabolic mirror M_2_. The incident light is non-polarized with a central wavelength of 650 nm and a full-width at half-maximum about 20 nm. Right panel: The entry of the LCP state (${|L}{\rm{\rangle }}$) into the visualization system allows for the acquisition of horizontal dimension information of the transparent flow field, while the RCP state (${|R}{\rm{\rangle }}$) provides vertical dimension information. Thus, the incidence of unpolarized light (UP) permits real-time observation of both dimensions. **b** Top-view and (**c**) perspective view of SEM images of the fabricated metasurface. The bottom inset shows the unit cell of the metasurface. The silicon nanopillar has a height of *h* and a period of *P*

Figure 1a presents a schematic of a non-invasive, fully two-dimensional transparent flow field visualization system, which can simultaneously generate two images by incorporating a designed photonic spin-decoupled metasurface array. One intensity image (${I}_{x}$) correlates with the horizontal density gradient $\partial \rho /\partial x$, following the relationship ${I}_{x}=H(\partial \rho /\partial x)$, where $H$ represents the transform function. The other image ${I}_{y}$ correlates with the vertical density gradient, following the relationship ${I}_{y}=H(\partial \rho /\partial y)$. Specifically, the first parabolic mirror (M_1_) serves to collimate the light beam. At each coordinate position (*x*, *y*) within the test region of the system, the directions of light rays are perturbed after passing through the transparent flow field. The second parabolic mirror (M_2_) performs a Fourier transform responsible for decomposing the disturbed optical wave into its constituent spatial frequency components ${\mathscr{F}}{\mathscr{(}}u,v)$. By inserting a photonic spin-decoupled metasurface array, represented by a Jones matrix $J(u,v)$, at the focal plane of M_2_, the spatial frequency spectrum can be independently tailored in both horizontal and vertical dimensions through two circular polarization channels. Following the inverse Fourier transformation operated by the focusing lens in front of the sensor, the metasurface-based system can simultaneously display the flow field in two dimensions, providing an intuitive visual image with functionalities unattainable by traditional optics.

The metasurface array comprises four distinct subregions, each represented by a Jones matrix designated as ${J}_{11}$, ${J}_{12}$, ${J}_{21}$, and ${J}_{22}$. Figure 1b, c show the scanning electron microscope (SEM) images of the metasurface, which is fabricated using a sequence of steps, including electron beam lithography, film deposition, lift-off, dry etching, and mask removal (see Materials and Methods section). In the system, when unpolarized light transmits through the metasurface array, its LCP and RCP components are subjected to distinct phase modulations and converted into cross-polarization states. In this process, the metasurface serves two roles: first, as a spatial filtering function $S(u,v)$: for LCP incident light, the metasurface acts as a step function in the $u$-direction, extracting flow field information in the horizontal dimension; similarly, for RCP incident light, it filters spatially in the $v$-direction to capture flow field information in the vertical dimension. Second, after spatial filtering, the information from the two dimensions still overlaps in space. Therefore, the second function of the metasurface is to decouple these two dimensions. In this process, the metasurface acts similarly to a beam splitter, separating the LCP and RCP components, which carry different flow field information. The general form of metasurface sub-arrays can be represented by the following equation:

1 $$J(u,v)=\left[\begin{array}{cc}{e}^{i{\varphi }_{{\rm{m}}}(u,v)} & {e}^{i{\varphi }_{{\rm{n}}}(u,v)}\\ {-{ie}}^{i{\varphi }_{{\rm{m}}}(u,v)} & {{ie}}^{i{\varphi }_{{\rm{n}}}(u,v)}\end{array}\right]{\left[\begin{array}{cc}1 & 1\\ i & -i\end{array}\right]}^{-1}$$

This matrix provides a general framework for mapping a pair of orthogonal states to two independent phase distributions ${\varphi }_{{\rm{m}}}$ and ${\varphi }_{{\rm{n}}}$, which can be utilized for manipulating wave vectors in Fourier space. In the design of metasurface devices, two phase control mechanisms are simultaneously employed: propagation phase modulation^29^ and geometric phase modulation.^30,45,46^ The anisotropic metasurface unit cells exhibit birefringent phase shifts along their major and minor axes, providing propagation phase modulation, while geometric phase modulation is achieved by altering the orientation angles of these unit cells. The eigenvalues and eigenvectors of the Jones matrix dictate the necessary birefringent phase shifts and fast-axis orientation angles for the nanopillars at each coordinate (*x*, *y*). Therefore, as long as the metasurface unit cells can meet these phase requirements, the desired optical functionalities can be realized.

**Fig. 2:**
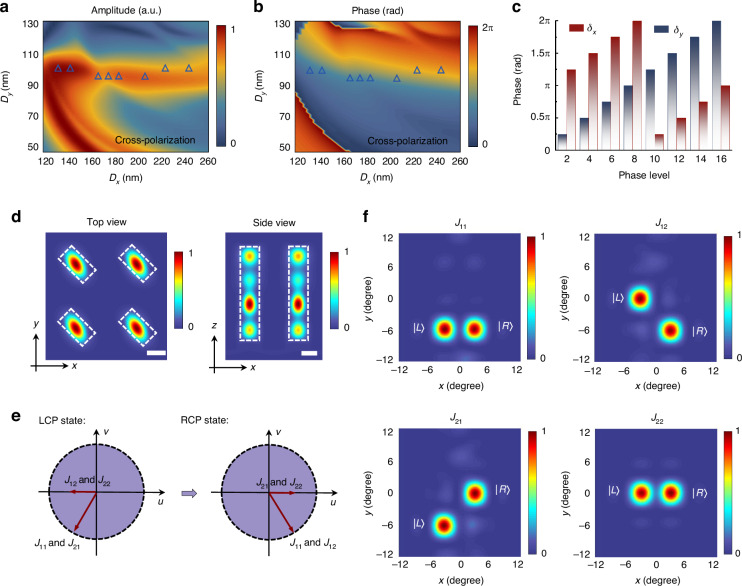
Design and functional verification of dielectric metasurface. **a** Amplitude and (**b**) phase retardation of cross-polarization after transmission through a periodic nanopillar array (*h* = 500 nm). The operational wavelength of the metasurface device is designed to be 650 nm. *D*_*x*_ and *D*_*y*_ represent the length and width of the cross-section of the nanostructure. Triangular symbols in the figure denote the selected nanopillars. **c** Calculated birefringent phase shifts, *δ*_*x*_ and *δ*_*y*_, for different phase levels. Each distinct phase level is associated with a specific nanostructure, where each nanostructure serves the role of a birefringent nano-waveplate. **d** Simulated energy density of a representative nanostructure under circularly polarized light illumination. Top and side views of the normalized energy density in an infinitely periodic array. The dashed white lines depict the boundaries of the nanopillars. Scale bars represent 100 nm. **e** The collaborative mechanism of the different metasurface sub-arrays. The red lines represent the wave vectors provided by different metasurface sub-arrays for LCP and RCP light. **f** Normalized far-field intensity distribution of each metasurface sub-array under unpolarized plane wave incidence

The metasurface is composed of rectangular silicon nanopillars on a transparent substrate, with each nanopillar designed to have a uniform height of *h* = 500 nm and organized in a square array with a lattice constant of *P* = 400 nm. By establishing a mapping between the parameters of nanostructures and polarization conversion efficiencies (Fig. 2a), as well as phase shifts (Fig. 2b), we can select the required nanostructures. Figure 2c shows the selected nanopillars with a birefringent phase difference of π and the capability to modulate phase covering 0-2π, laying the foundation for designing spin decoupling functionality. When light waves pass through each silicon nanopillar, they are primarily concentrated inside the pillars, which act as weakly coupled low-quality factor resonators (Fig. 2d). The entire metasurface device has dimensions of 4 × 4 mm² and is composed of 100 million silicon nanopillars. We define the polarization conversion efficiency as the ratio of the transmitted cross-polarized light power to the total power of the incident circularly polarized light. Measurement results indicate that the metasurface device, at the operating wavelength of 650 nm, has a polarization conversion efficiency of 20.4% for converting LCP into RCP, and an efficiency of 19.5% for converting RCP into LCP. The relatively low efficiency is mainly attributed to device reflection, losses, and fabrication errors.

Figure 2e illustrates the wavevectors provided by each sub-array of the metasurface for different polarization states of light. The metasurface sub-arrays, ${J}_{11}$ and ${J}_{21}$, provide the wavevector for the incident LCP state as: ${\vec{k}}_{{LCP}}=-[{k}_{0}\sin ({3}^{\circ })\hat{u}+{k}_{0}\sin ({6}^{\circ })\hat{v}]$, where ${k}_{0}=2\pi /\lambda$. Meanwhile, ${J}_{12}$ and ${J}_{22}$ provide the wavevector ${\vec{k}}_{{LCP}}=-{k}_{0}\sin ({3}^{\circ })\hat{u}$, acting as a step function in the *u*-direction. Simultaneously, the sub-arrays ${J}_{11}$ and ${J}_{12}$ provide the wavevector for the incident RCP state as: ${\vec{k}}_{{RCP}}=[{k}_{0}\sin ({3}^{\circ })\hat{u}-{k}_{0}\sin ({6}^{\circ })\hat{v}]$, while ${J}_{21}$ and ${J}_{22}$ provide the wavevector ${\vec{k}}_{{RCP}}={k}_{0}\sin ({3}^{\circ })\hat{u}$, acting as a step function in the *v*-direction. Figure 2f shows simulation results for the functionality of each sub-region of the metasurface, which are consistent with our designs depicted in Fig. 2e. More detailed descriptions of the metasurface design are provided in Section 1 of the supplementary materials.

### Visualization and quantitative measurement of temperature fields

**Fig. 3:**
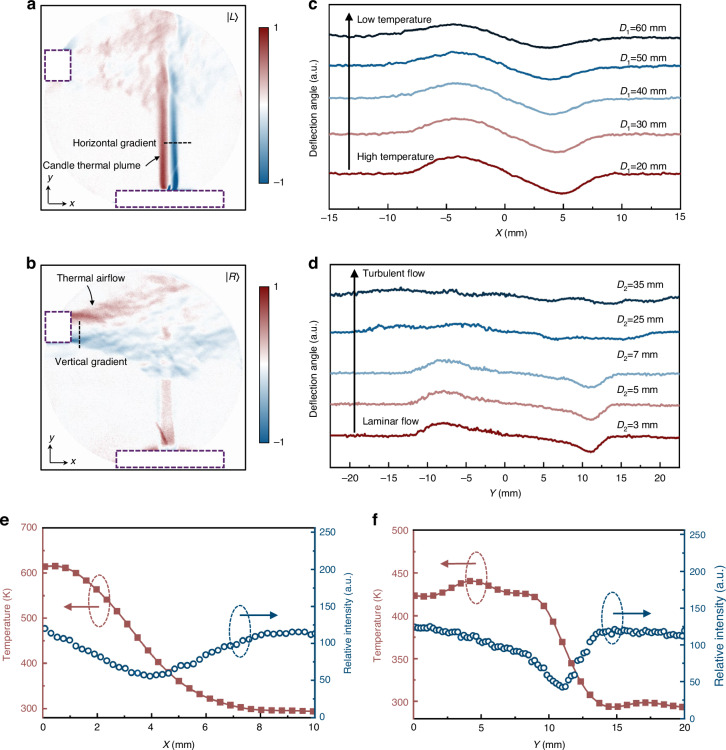
Visualization and quantitative analysis of temperature fields. **a**, **b** Full two-dimensional flow field images obtained simultaneously through a single shot, including the horizontal gradient image (**a**) and the vertical gradient image (**b**). The purple dashed box indicates the positions of the candle and heat gun. The displayed images present effective flow field information obtained by subtracting reference images captured in the absence of the flow field. **c**, **d** Light deflection induced by temperature variations in the flow field, with (**c**) corresponding to the thermal plume of the candle and (**d**) to the thermal airflow of the heat gun. *D*_1_ represents the distance from the candle wick to the measured thermal plume, and *D*_2_ represents the distance from the nozzle of the heat gun to the measured hot air. **e**, **f** Reconstructed results of the temperature distribution for the candle’s thermal plume (**e**) and the heat gun’s thermal airflow (**f**)

To demonstrate the superiority of the proposed flow field visualization method, we first conduct experiments within temperature fields, featuring a heat gun positioned in the upper left and a candle in the lower right. The laboratory ambient temperature is maintained at 293 K. The thermal plume from the candle and the thermal airflow from the heat gun, which are entirely invisible to the naked eye, are clearly visible using our system. Figure 3a, b present the flow field images of two orthogonal dimensions obtained simultaneously through a single shot. The thermal plume of the candle exhibits significant temperature variations in the horizontal direction, with minimal changes in the vertical direction (Fig. 3a). In contrast, the thermal airflow from the heat gun shows significant temperature variations in the vertical direction, with minimal changes in the horizontal direction (Fig. 3b), before it transitions into turbulence.

Our approach not only enables clear visualization of transparent flow fields in a non-invasive manner, providing real-time observation with the naked eye, but also captures extensive information within the flow field, such as the evolution from laminar to turbulent flow, patterns of temperature change, and even specific temperature data. By employing the standard photometric method (Section 2 of the supplementary materials), we can obtain the distribution of light deflection angles induced by temperature variations in the flow field (Fig. 3c and d). The thermal plume of the candle maintains a laminar flow state over a considerable propagation distance, with temperatures gradually decreasing in the vertical direction; in contrast, the heat gun’s thermal airflow starts as laminar but quickly transitions to turbulent as the propagation distance increases. Assuming an axially symmetric distribution in the flow field, we utilize the Abel inverse transformation to obtain further detailed information on the temperature distribution. Figure 3e displays the radial temperature distribution at a distance of 50 mm from the wick, where the central temperature reaches 614 K, and measurements from a thermocouple wire show 589 ± 3.6 K. Figure 3f displays the radial temperature distribution at a distance of 5 mm from the nozzle of the heat gun, where the central temperature is 423 K, with thermocouple measurements at 408 ± 2.1 K. The reconstructed results show high consistency with the actual measurements, with relative errors of 4.2% and 3.7%, respectively, demonstrating the excellent reliability of our method.

### Visualization and quantitative measurement of gas flow fields

Gas leak detection is crucial for ensuring the safe operation of oil refineries and petrochemical plants, as it can promptly identify potential hazards and prevent large-scale casualties and environmental pollution. Early detection and rapid response significantly reduce the accidents and losses caused by leaks. Although conventional technologies such as pressure measurement, flow monitoring, and acoustic sensing provide some level of surveillance, they often struggle to precisely pinpoint leak locations and find it challenging to detect minor leaks. The metasurface-based system offers an efficient solution for gas leak detection, allowing for direct visualization, accurate localization of leaks, and continuous monitoring, and is particularly effective for detecting small-scale gas emissions.

**Fig. 4:**
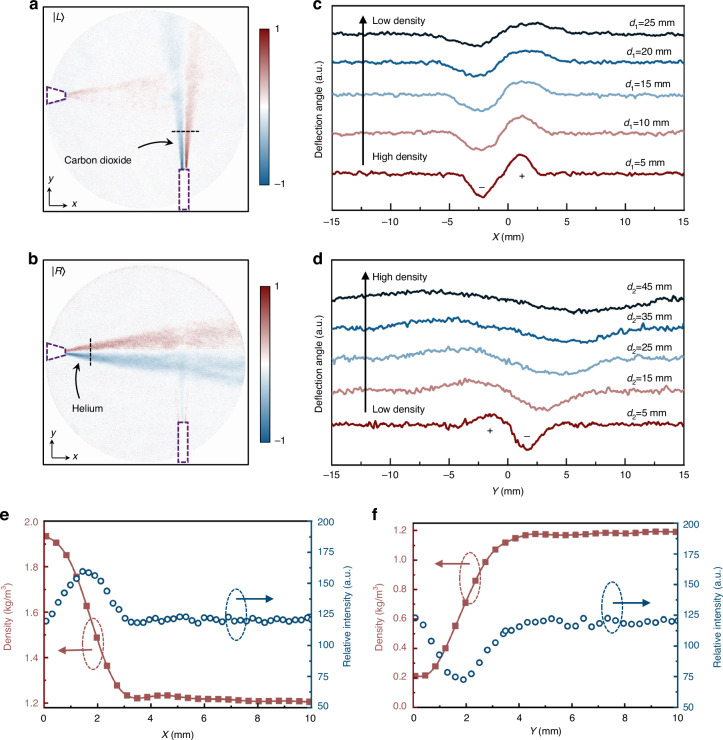
Visualization and quantitative analysis of transparent gas flow. **a**, **b** Single-shot acquisition of the horizontal and vertical dimensions of the gas flow field: (**a**) CO_2_ jet field, (**b**) He jet field. The purple dashed box indicates the position of the gas outlet. **c**, **d** Light deflection caused by density variations in the flow field, with (**c**) corresponding to the CO_2_ jet field, and (**d**) corresponding to the He jet field. *d*_1_ and *d*_2_ represent the distances from the gas emission port to the measured flow field. The ‘+’ and ‘-’ symbols represent light deflection to the left (up) and right (down), respectively. **e**-**f** Radial density distribution of CO_2_ (**e**) and He (**f**) at a position 5 mm from the gas outlet

To showcase the advantages of the metasurface-based visualization system in the field of gas leak detection, carbon dioxide (CO_2_) and helium (He) jet fields are used as targets. These gases are invisible to the naked eye, yet the visualization system can vividly display the flow field information of both gases (Fig. 4a, b), revealing their diffusion patterns. Because carbon dioxide has a higher density than air, while helium has a lower density, they exhibit opposite signs in light deflection angles (Fig. 4c and d), a phenomenon confirmed by our experiments. Through the Abel inversion, we can calculate the flow field densities at specific locations, thereby further identifying the types of gases leaking, an achievement not possible with conventional acoustic sensing technologies. As shown in Fig. 4e, f, the density reconstructions at a distance of 5 mm from the gas outlet show that the central densities of the CO_2_ and He gas flows are 1.934 kg·m^-3^ and 0.205 kg·m^-3^, respectively. These values are close to the densities of pure CO_2_ and pure He, which are 1.977 kg·m^-3^ and 0.179 kg·m^-3^, respectively, indicating minimal mixing of these two gases with air at this stage. Additionally, our technique enables the detection of minor gas leaks, as evidenced by our experiments where a leak as small as 0.13 mL·s^-1^ still clearly reveals the flow field information (Fig. S5 in the supplementary materials).

### Visualization of various fluid physical phenomena

**Fig. 5:**
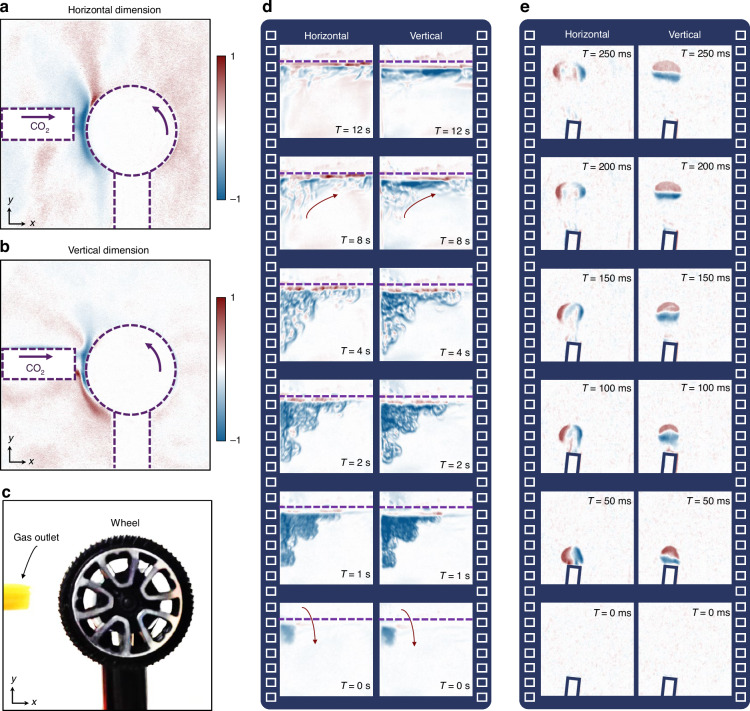
Visualization of various fluid physical phenomena. **a**, **b** Magnus effect and (**c**) its corresponding experimental setup. **d** Phenomenon of thermal convection in liquids. **e** Spatiotemporal evolution of a butane gas cluster

We further demonstrate the application of the proposed imaging method in the study of fluid physical phenomena. Figure 5a, b illustrate the flow field structures associated with the Magnus effect. In our experiments, a wheel with a diameter of 3 cm rotates counterclockwise at a speed of 500 revolutions per minute and is positioned in a CO_2_ gas stream (Fig. 5c). The horizontal dimension image shows that the flow field movement in the upper left of the wheel is significantly obstructed (Fig. 5a), resulting in a reduction in flow speed at that location. From the vertical dimension image, it is more evident that the wheel’s rotation induces fluid rotation (Fig. 5b), increasing the flow speed below the wheel. According to Bernoulli’s principle, the increase in fluid speed below the wheel leads to a decrease in pressure, while the reduction in fluid speed in the upper left of the wheel leads to an increase in pressure. As expected, a pressure difference forms around the wheel, generating a downward Magnus force.

In addition, our visualization method allows real-time observation of the convection phenomenon and the instantaneous evolution of flow field structures. In our experiment, we introduce 60 °C hot water into 10 °C cold water and capture the evolution of convection in real-time (Fig. 5d). At *T* = 0 s, the image shows the moment when hot water is just added to the cold water, with the dark blue area representing the region occupied by the newly introduced hot water. Between 0-2 s, the hot water, driven by gravitational potential energy, continues to fall into the cold water and diffuses to the right. At *T* = 2 s, the absence of strong contrast in light intensity indicates that the downward kinetic energy of the hot water is nearly zero. From 2–12 s, due to the lower density of hot water compared to cold water, the hot water begins to flow upward while the cold water flows downward, forming convection currents. At *T* = 12 s, this stage of the convection process is essentially complete. The hot water has reached the surface, with cold water below it. At this point, the vertical dimension reveals strong light intensity contrast only at the surface, indicating a clear temperature gradient distribution at the surface. However, the horizontal dimension shows that the light intensity contrast is also concentrated at the surface but distributed more randomly, suggesting that the horizontal state has not yet stabilized, and local micro-convection still exists.

Besides the observation of the convection phenomenon, we also conducted an experiment to capture the spatiotemporal evolution of the butane gas cluster with a high-speed camera at a rate of 500 frames per second (FPS), as shown in Fig. 5e. Initially, when a small amount of butane gas is released from the gas cylinder, it acquires initial momentum and moves upward. At T = 100 ms, both the horizontal and vertical dimensions reveal a certain degree of symmetry in the flow field structure, with the gas cluster exhibiting an irregular spherical shape. As the propagation distance increases, the horizontal flow field structure expands while the vertical flow field structure remains almost unchanged, transforming the gas cluster from a sphere into an ellipsoid. The combined effects of viscous drag from the surrounding air and turbulent diffusion primarily cause this phenomenon. For rapidly changing transparent flow fields (such as intense turbulence), the image sensor at the backend can be upgraded to a camera with a higher frame rate to capture rapidly evolving details.

### 3D morphological reconstruction of transparent solids

**Fig. 6:**
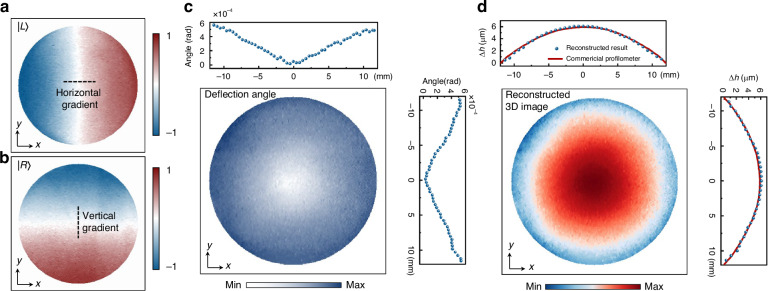
3D morphological reconstruction of transparent solids. **a**, **b** A single-shot captured two images of a planoconvex lens under test. **c** Light deflection caused by phase accumulation within the lens. **d** 3D morphological reconstruction result of the lens. The red solid line and the dark blue dots represent the measurement results from the scan-type profilometer and our reconstruction results, respectively

It is noteworthy that the proposed metasurface system is not only applicable to the visualization of flow fields but also suitable for retrieving the 3D morphology of transparent solids, such as various optical lenses. Figure 6a, b display images of the planoconvex lens under test, obtained from a single capture in two dimensions. By synthesizing the vectors from both dimensions, the overall deflection angle distribution of the planoconvex lens can be determined (see Fig. 6c and Section 2.5 of the Supplementary Text). The thickness is then calculated based on the distribution of light deflection angles and the refractive index of the material constituting the planoconvex lens. Figure 6d presents the 3D image of the lens. To validate our results, we conducted ground-truth measurements using a commercial profilometer. Our reconstruction results (deep blue dots) are highly consistent with the profilometer measurements (red solid lines). In the reconstruction results, the average height difference between the center and the edges is Δ*h̅* = 6.12 μm, while the measurement result from the commercial profilometer is 5.95 μm, with a relative error of ~2.9%. This single-shot, non-contact measurement method is particularly suited for applications requiring rapid, high-precision inspections and measurements, or where high-resolution depth information is needed.

## Discussion

In summary, we have developed a non-invasive visualization platform for transparent flow fields using photonic spin-decoupled metasurfaces. Utilizing the naturally decoupled LCP and RCP components from incident unpolarized light, this system can simultaneously capture flow field information in two orthogonal dimensions in a single shot. This capability facilitates real-time, quantitative reconstruction of multidimensional physical parameters. With this metasurface-based visualization platform, we experimentally demonstrate diverse applications, including temperature field mapping, gas leak detection, visualization of various fluid physical phenomena, and 3D morphology reconstruction of transparent phase objects. The platform enables direct observation of turbulence, laminar flow, and vortices in transparent fluids within small-scale environments, promoting a deeper understanding of the physical nature of these phenomena. Additionally, this approach shows potential in industrial design and vision, such as optimizing streamlined automobiles and aircraft.

## Materials and methods

### Metasurface fabrication

The fabrication process of the designed metasurface device is mainly accomplished through electron beam lithography, thin film deposition, and reactive ion etching technology (Supplementary Fig. S1). Specifically, the process begins with a Silicon-on-Sapphire (SoS) wafer, consisting of a 500 nm thick monocrystalline silicon layer and a 460 μm thick sapphire substrate. The SoS wafer undergoes O_2_ plasma treatment, followed by vapor-coating a layer of hexamethyldisilazane (HMDS) to improve adhesion. Subsequently, a 180 nm thick layer of positive e-beam resist (ZEP520A) is spin-coated onto the silicon film at 4000 rpm, followed by a 3 min baking at 180 °C on a hotplate. To eliminate the charge accumulation effects, the surface of the sample is further spin-coated with a layer of conductive adhesive (ARPC5090). The designed patterns are defined via an electron beam lithography system (Elionix, ELS-F125), and a solution of ZEDN50 (n-Amylacetate) is employed to develop the photoresist. Then, a 30 nm thick aluminum (Al) layer is deposited on the photoresist by employing an electron beam evaporator (SKY, DZS500). Subsequently, a lift-off process is performed in NMP (n-methyl-pyrrolidone) to generate the hard mask, enabling the developed patterns in the resist layer to be transferred to the Al layer. To form the silicon nanopillars, inductively coupled plasma reactive ion etching (ICP-200, Tailong Electronics) is carried out with a mixture of C_4_F_8_ and SF_6_ gases at a ratio of 2.5 (C_4_F_8_/SF_6_). The process involves applying a bias radiofrequency power of 40 W and an ICP power of 500 W and maintaining a pressure of 13 mTorr. Finally, the hard mask is removed with the stripping solution (Aluminum Etchant Type A, Sigma-Aldrich).

## Supplementary information

Supplementary Inforamtion for Non-Invasive and Fully Two-Dimensional Quantitative Visualization of Transparent Flow Fields Enabled by Photonic Spin-Decoupled Metasurfaces

## Data Availability

All the data in this study are provided within the paper and its supplementary information. The data of this study are available from the corresponding author upon reasonable request.
