# Supplementary material for: Non-invasive and fully two-dimensional quantitative visualization of transparent flow fields enabled by photonic spin-decoupled metasurfaces
Source: Light Sci Appl. 2025 Mar 5;14:113. doi: 10.1038/s41377-025-01793-2 (PMC11882825; doi:10.1038/s41377-025-01793-2)
Supplement: Supplementary file 1 — Supplementary Inforamtion for Non-Invasive and Fully Two-Dimensional Quantitative Visualization of Transparent Flow Fields Enabled by Photonic Spin-Decoupled Metasurfaces [file 41377_2025_1793_MOESM1_ESM.pdf]

Supplementary Materials for "Non-Invasive and Fully Two-Dimensional Quantitative Visualization of Transparent Flow Fields Enabled by Photonic Spin-Decoupled Metasurfaces"

Qingbin Fan[1,2,3#], Peicheng Lin[1,3#], Le Tan[1,2,3#], Chunyu Huang[1], Feng Yan[2*], Yanqing Lu[1,3*] and Ting Xu[1,3,4*]

1. National Laboratory of Solid-State Microstructures and Collaborative Innovation Center of Advanced Microstructures, Nanjing University, Nanjing 210093, China
2. School of Electronic Sciences and Engineering, Nanjing University, Nanjing 210093, China
3. College of Engineering and Applied Sciences and Key Laboratory of Intelligent Optical Sensing and Manipulation, Ministry of Education, Nanjing University, Nanjing 210093, China
4. School of Materials Engineering, Jiangsu University of Technology, 213001, Changzhou, China

# These authors contributed equally to this work.

Email: fyan@nju.edu.cn; yqlu@nju.edu.cn; xuting@nju.edu.cn

Supplementary Text

1. Details on Metasurface Device Design

As described in the main text, the nanophotonic device we have designed comprises four distinct sub-regions, each of which can be represented by Jones matrices labeled as J_{11}, J_{12}, J_{21}, and J_{22}, respectively. These sub-regions serve different functions and collaboratively achieve powerful optical manipulation capabilities. Here, we provide a detailed introduction to the photonic spin-decoupled metasurface. Subsequently, we elaborate on the collaboration mechanism of the four sub-regions.

1.1 Photonic spin-decoupled metasurface

The metasurface device is required to attain independent phase modulation of the LCP and RCP input states, achieving a fully decoupled output. Notably, most transparent fluid flows in nature respond identically to LCP and RCP light. Assuming the incident polarization state is $|L\rangle$, the metasurface imparts a phase profile of $\varphi_{\mathrm{m}}(u,v)$ to the incident light. If the incident polarization state is $|R\rangle$, the metasurface imparts a different phase profile, $\varphi_{\mathrm{n}}(u,v)$, to the incident light. Such a device can be described by the following Jones matrix [1-3]:

$$J(u,v) = \frac{1}{2}\begin{bmatrix} e^{i\varphi_{\mathrm{m}}(u,v)} + e^{i\varphi_{\mathrm{n}}(u,v)} & ie^{i\varphi_{\mathrm{n}}(u,v)} - ie^{i\varphi_{\mathrm{m}}(u,v)} \\ ie^{i\varphi_{\mathrm{n}}(u,v)} - ie^{i\varphi_{\mathrm{m}}(u,v)} & -e^{i\varphi_{\mathrm{m}}(u,v)} - e^{i\varphi_{\mathrm{n}}(u,v)} \end{bmatrix} \tag{S1}$$

This matrix provides the general form of photonic spin-decoupled metasurfaces. Assuming that the birefringence phases of the anisotropic nanostructure are δ_x and δ_y, with an orientation angle of θ. By computing the Jones matrix $J(u,v)$, we can deduce the phase shifts as follows:

$$\delta_x(u,v) = [\varphi_m(u,v) + \varphi_n(u,v)]/2 \tag{S2a}$$

$$\delta_y(u,v) = [\varphi_m(u,v) + \varphi_n(u,v)]/2 - \pi \tag{S2b}$$

and the rotation angle as:

$$\theta(u,v) = [\varphi_m(u,v) - \varphi_n(u,v)]/4 \tag{S2c}$$

1.2 Decoupling of flow field information in horizontal and vertical dimensions

Based on the mentioned photonic spin-decoupling method, we can design four sub-regions (J_{11}, J_{12}, J_{21}, J_{22}) to achieve the decoupling of 2D flow field information through the synergy among these sub-regions. In the main text, we have provided the general form of the Jones

matrix and the specific functions of each metasurface. Here, we present the specific form of each metasurface sub-array:

$$J_{11}(u,v) = \begin{bmatrix} e^{i[\varphi(u)+\varphi(v)]} & e^{i[\varphi(v)-\varphi(u)]} \\ -ie^{i[\varphi(u)+\varphi(v)]} & ie^{i[\varphi(v)-\varphi(u)]} \end{bmatrix} \begin{bmatrix} 1 & 1 \\ i & -i \end{bmatrix}^{-1} \quad \text{(S3a)}$$

$$J_{12}(u,v) = \begin{bmatrix} e^{i\varphi(u)} & e^{i[\varphi(v)-\varphi(u)]} \\ -ie^{i\varphi(u)} & ie^{i[\varphi(v)-\varphi(u)]} \end{bmatrix} \begin{bmatrix} 1 & 1 \\ i & -i \end{bmatrix}^{-1} \quad \text{(S3b)}$$

$$J_{21}(u,v) = \begin{bmatrix} e^{i[\varphi(u)+\varphi(v)]} & e^{-i\varphi(u)} \\ -ie^{i[\varphi(u)+\varphi(v)]} & ie^{-i\varphi(u)} \end{bmatrix} \begin{bmatrix} 1 & 1 \\ i & -i \end{bmatrix}^{-1} \quad \text{(S3c)}$$

$$J_{22}(u,v) = \begin{bmatrix} e^{i\varphi(u)} & e^{-i\varphi(u)} \\ -ie^{i\varphi(u)} & ie^{-i\varphi(u)} \end{bmatrix} \begin{bmatrix} 1 & 1 \\ i & -i \end{bmatrix}^{-1} \quad \text{(S3d)}$$

where $\varphi(u) = -k_0 \sin(\theta_1)u$ and $\varphi(v) = -k_0 \sin(\theta_2)v$. The phase profiles $\varphi(u)$ and $\varphi(v)$ provide specific wave vector $\boldsymbol{k}$ = (k_u, k_v) along the horizontal and vertical directions, respectively. The wave vectors provided by each metasurface sub-array are determined based on the following three factors: (i) Spatial filtering: for LCP and RCP light, the entire device behaves as a step function along the horizontal and vertical directions, respectively; (ii) Spatially decoupling the flow field information in the horizontal and vertical dimensions, ensuring that the flow field images in the two dimensions do not overlap; (iii) Designing the optical system for off-axis imaging to eliminate the interference of zeroth-order transmitted light, thereby ensuring high-quality data acquisition. Assuming that the focal length of the second parabolic mirror is f and its diameter is D, the magnitude of the provided wave vector can be determined by the following expression:

$$k_u \geq \frac{k_0 D}{2f} \quad \text{(S4a)}$$

$$k_v \geq \frac{k_0 D}{f} \quad \text{(S4b)}$$

Here, $k_0 = 2\pi/\lambda$ is the magnitude of the wave vector in free space. Based on Eq. 4a and Eq. 4b, the values of θ_1 and θ_2 are designed to be 3° and 6°, respectively.

To further comprehend how the Jones matrix for each sub-region controls the output, we can simply employ the conventional Jones matrix formalism, which relates the input electric field to the output polarization as $|E_{out}\rangle = J|E_{in}\rangle$. When the incident light is LCP state, the output for each metasurface subarray is as follows:

$$|E_{11}\rangle = e^{i[\varphi(u)+\varphi(v)]}|R\rangle \quad \text{(S5a)}$$

$$|E_{12}\rangle = e^{i\varphi(u)}|R\rangle \quad \text{(S5b)}$$

$$|E_{21}\rangle = e^{i[\varphi(u)+\varphi(v)]}|R\rangle \quad \text{(S5c)}$$

$$|E_{22}\rangle = e^{i\varphi(u)}|R\rangle \tag{S5d}$$

When the incident light is RCP state, the output for each metasurface subarray is as follows:

$$|E_{11}\rangle = e^{i[\varphi(v)-\varphi(u)]}|L\rangle \tag{S6a}$$

$$|E_{12}\rangle = e^{i[\varphi(v)-\varphi(u)]}|L\rangle \tag{S6b}$$

$$|E_{21}\rangle = e^{-i\varphi(u)}|L\rangle \tag{S6c}$$

$$|E_{22}\rangle = e^{-i\varphi(u)}|L\rangle \tag{S6d}$$

From Eq. 5a to Eq. 5d, it can be seen that, as expected, the optical waves passing through subarrays J_{11} and J_{21} undergo identical phase modulation, providing the desired wave vector. On the other hand, the waves passing through subarrays J_{12} and J_{22} are modulated in the horizontal direction. Thus, the entire metasurface acts as a step function along the u-direction, achieving the spatial frequency filtering function. Therefore, the incident LCP state enables the detection of flow field information in the horizontal dimension. Similarly, the incident RCP state enables the detection of flow field information in the vertical dimension. Furthermore, Fig. S2 presents additional simulation validations of the functionalities of each subarray, and the results are consistent with our expectations.

1.3. Design and simulation of subwavelength nano-waveplate element

As mentioned above, achieving photonic spin-decoupling in the metasurface requires a coordinated modulation mechanism of both the propagation phase and the geometric phase. The propagation phase designs rely on the nanopillar's geometry, enabling the metasurface to apply distinct spatial phase profiles on two orthogonal, linear polarizations [1]. On the other hand, the geometric phase designs are exclusively governed by the angular orientation (θ) of the nanopillar, allowing for the imposition of phase profiles that are equal in magnitude but opposite in sign ($\varphi=\pm2\theta$) on a pair of orthogonal circular polarizations [4, 5]. According to Eq. S2, it is crucial to search for a set of birefringent nanostructures that fulfill specific conditions. These conditions encompass: (a) The chosen nanostructures all serve as ideal half-wave plates, where the coefficients of the complex amplitude for transmitted orthogonal linearly polarized light are equal, and the phase difference is π, namely $t_x = t_y$ and $\delta_x = \delta_y + \pi$. (b) The propagation phase (δ_x and δ_y) of this set of structures should span the range from 0 to 2π.

In this work, the material forming the nanopillars is monocrystalline silicon. The choice

of monocrystalline silicon as the material component is primarily attributed to its high refractive index, low loss, and compatibility with CMOS manufacturing processes. The first task involves meticulously performing parameter scans on the long and short axes (D_x and D_y) of the nanostructure, and creating a library to establish the relationship between the optical response (*e.g.,* amplitude and phase) of the nanostructure and its dimensions. Based on the complex amplitude information of two orthogonal linearly polarized transmissions through the nanostructure, we can calculate the polarization conversion efficiency and phase distribution across the entire parameter space (Figs. 2a and 2b). According to Eq. S2, we selected a set of nanostructures as the basic building blocks of metasurface devices. Fig. S3 illustrates the complete design process of the metasurface device.

2. Reconstruction algorithm

In our work, the reconstruction process primarily involves several key components, including the standard photometry method, the Abel inversion transform (based on the assumption of axial symmetry), the Gladstone-Dale relationship, and the ideal gas equation, as shown in Fig. S4. Below, we will provide a detailed description of these aspects.

2.1 Standard photometry method

In the first step, the standard photometry method employs a standard lens with known parameters as a reference to measure the deflection angles of light rays within the designed imaging system and establishes a correlation between these deviation angles and image grayscale values. Specifically, a light ray passing through an arbitrary point on the lens, denoted as r, will undergo refraction at an angle ε:

$$\tan\varepsilon = \frac{r}{f} \tag{S7}$$

The calibration lens must have an extremely high F-number, meaning a focal length significantly greater than the lens diameter. This is essential to generate refraction angles within the region of interest, allowing for the precise quantification of light ray deflection caused by flow field disturbances. In this work, we used a customized lens with a radius of 12.7 mm and a focal length of 20 m, allowing for the quantification of refraction angles up to ~131 arc-seconds (6.35×10^{-4} rad). In this case, under the small-angle approximation, Eq. 7 evolves to: $\varepsilon \approx \frac{r}{f}$. The calibration lens refracts parallel light and focuses it to a location beyond the position of the metasurface device. As this beam of light passes through the metasurface device, it undergoes spatial modulation due to the refraction angle within the lens, resulting in the intensity gradient observed on the image sensor. The intensity of each grayscale pixel in the lens image can be quantified to the refractive angle in the corresponding dimension.

The calibration starts with determining the average background pixel intensity of the image. This identical intensity value is subsequently identified within the calibration lens image. The distance of this point from the center of the lens, measured along the horizontal or vertical diameter, depending on the current dimension, is defined as r_0. It represents the pixel intensity value in the image when unaffected by flow field disturbances and serves as the baseline for all

other measurements. According to Eq. 7, the angle of light deflection corresponding to this point can be obtained as ε_0.

Therefore, the refraction angle at any point in the image affected by flow field disturbances can be quantified by first identifying the position within the calibration lens image that shares the same pixel intensity as the point of interest. This point in the lens image has a distance of r from the lens center, and the angle of light refraction is denoted as ε. The deflection angle ε_d at the corresponding point in the image affected by flow field disturbances is equivalent to the relative refraction angle between positions r and r_0 in the calibration lens image, namely:

$$\varepsilon_d = \varepsilon - \varepsilon_0 = \frac{r - r_0}{f} \tag{S8}$$

2.2 Abel inversion transform

The second step is to derive the refractive index distribution of the flow field based on the deflection angles corresponding to each point in the captured image. Several experiments in the main text involve cases where the refractive index exhibits an axisymmetric distribution. For an axisymmetric medium, Abel inversion formulas can be employed to reconstruct the refractive index distribution from its one-dimensional projections [6]. Assuming the refractive index distribution function is $\delta = (\eta/\eta_0 - 1)$, where η represents the local refractive index, and η_0 is the refractive index of the surrounding medium. Taking the horizontal dimension as an example, the relationship between the deflection angle of light rays and the refractive index distribution is as follows:

$$\varepsilon(x) = 2x \int_x^R \frac{\partial \delta}{\partial r} \frac{dr}{\sqrt{r^2 - x^2}} \tag{S9a}$$

$$\delta(r) = -\frac{1}{\pi} \int_r^\infty \varepsilon(x) \frac{dx}{\sqrt{x^2 - r^2}} \tag{S9b}$$

Eqs. S9a and S9b correspond to the forward and inverse Abel transformations, where Eq. S9a addresses the direct problem of evaluating angular deflection, while Eq. S9b deals with the inverse problem.

The Abel inverse transformation can be solved using a Fourier analysis-based approach. The unknown radial distribution, $\delta(r)$, is expanded into a series of cosine functions, and the amplitudes of these functions are calculated by least-squares-fitting of the Abel-transformed series to the measured data $\varepsilon(x)$. The resulting amplitudes are then inserted into the expansion of $\delta(r)$, producing the desired distribution.

Specifically, the unknown refractive index distribution $\delta(r)$ is expanded into a Fourier series as:

$$\delta(r) = \sum_{n=N_l}^{N_u} A_n \delta_n(r) \tag{S10}$$

where A_n represents the unknown amplitudes and $\delta_n(r)$ can be a set of cosine functions as follows:

$$\delta_0(r) = 1$$

$$\delta_n(r) = 1 - (-1)^n \cos(n\pi \frac{r}{R}) \tag{S11}$$

Therefore, the Abel forward transform applied to the function $\delta(r)$ yields the following expression:

$$H(x) = 2\sum_{n=N_l}^{N_u} A_n \int_x^R \frac{\partial \delta_n}{\partial r} \frac{dr}{\sqrt{r^2 - x^2}} \tag{S12}$$

$$h_n(x) = \int_x^R \frac{\partial \delta_n}{\partial r} \frac{dr}{\sqrt{r^2 - x^2}} \tag{S13}$$

The amplitude A_n is an unknown quantity, and it can be considered that at $x = x_k$, the function $H(x_k)$ should approximate the measured values $\varepsilon(x_k)$. This statement can be expressed as a least-squares criterion:

$$\sum_{k=N_l}^{N_u} [H(x_k) - \varepsilon(x_k)]^2 \rightarrow Min \tag{S14}$$

The final amplitudes A_n are inserted into Eq. S10, thus yielding the final distribution $\delta(r)$.

2.3 Gladstone-Dale relation

Once the refractive index is obtained, further information about the density distribution of transparent medium can be derived using the Gladstone-Dale relationship. The Gladstone-Dale relationship describes the relationship between the refractive index (n) of a medium and its density (ρ). The general form of the Gladstone-Dale relationship is expressed as:

$$n = k\rho + 1 \tag{S15}$$

Here, n represents the refractive index of the medium. k is the Gladstone-Dale constant, which is a medium-specific constant that characterizes how the refractive index changes with density. ρ stands for the density of the medium. The Gladstone-Dale constant for air is approximately k=2.23×10^{-4} m^3·kg^{-1}.

2.4 Derivation of temperature distribution

After obtaining the refractive index distribution for the candle flame scene, we can further deduce its temperature distribution. Based on the Gladstone-Dale relationship and the ideal gas equation, we can obtain the following expression:

$$T = \frac{n_0 - 1}{n - 1} T_0 \tag{S16}$$

Here, n_0 and T_0 represent the refractive index of air in the experimental environment and the ambient temperature. This formula is an approximation applicable to relatively low pressure and temperature conditions of ideal gases.

2.5 Calculation of total light ray deflection angle

The light ray direction can be represented as the vector $\vec{d} = (d_x, d_y, d_z)$. The angles θ_x, θ_y, and θ_{total} can be described as follows: θ_x is the angle between the projection of the light ray in the XZ plane and the Z-axis. θ_y is the angle between the projection of the light ray in the YZ plane and the Z-axis. θ_{total} is the total angle between the light ray and the Z-axis.

Using the tangent function: $\tan(\theta_x) = d_x/d_z$, $\tan(\theta_y) = d_y/d_z$. The total angle θ_{total} can be expressed using the vector components. The projection of $\vec{d}$ onto the XY plane is $\sqrt{d_x^2 + d_y^2}$. Therefore, we have: $\tan(\theta_{total}) = \sqrt{d_x^2 + d_y^2}/d_z$. Using the relationships for $\tan(\theta_x)$ and $\tan(\theta_y)$, we can rewrite this as:

$$\tan(\theta_{total}) = \sqrt{\tan^2(\theta_x) + \tan^2(\theta_y)} \tag{S17}$$

Through Eq. S17, the total deflection angle of the light ray after passing through the lens can be calculated, as depicted in Fig. 6 of the main text. Given the material's refractive index (n=1.5168), the thickness distribution across the entire lens can be further obtained.

2.6 Spatial Resolution

We obtained the modulation transfer function (MTF) curve by experimentally measuring the system's response across different spatial frequencies. As shown in Fig. S6, the measured system resolution reaches 1.12 lp·mm^{-1}, equivalent to a line width of 446 μm. Further enhancement of spatial resolution can be achieved by optimizing the optical components in the system, such as incorporating higher-precision parabolic mirrors or improving the imaging lens at the front end of the image sensor.

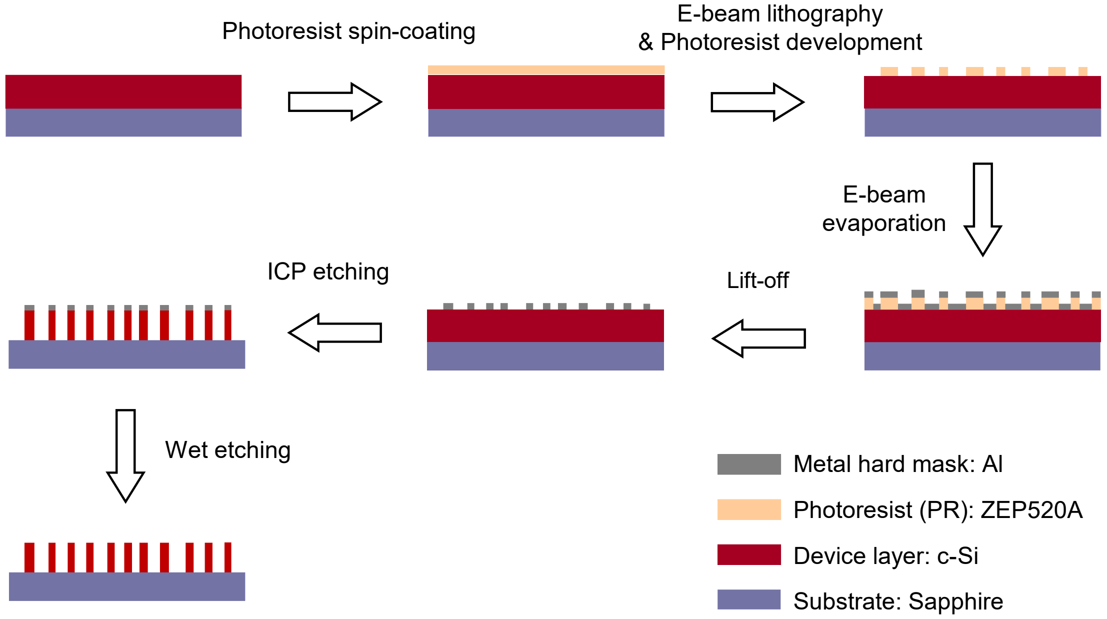

Figure S1 | Schematic illustration of the metasurface fabrication. The fabrication process of the designed metasurface device is mainly accomplished through electron beam lithography, thin film deposition, and reactive ion etching technology.

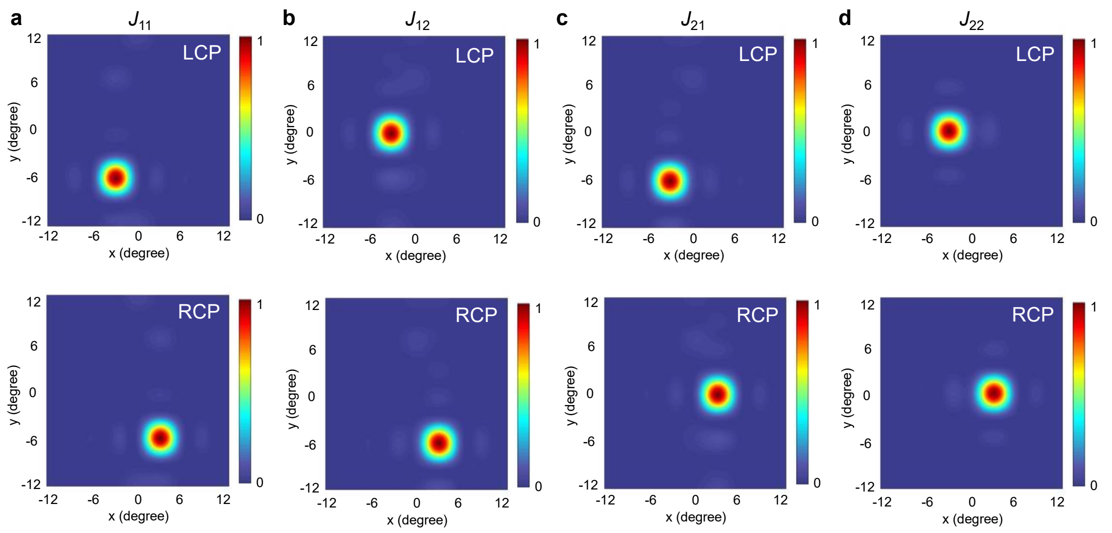

Figure S2 | Normalized far-field intensity images of simulated metasurface sub-regions. Panels (**a-d**) respectively display the far-field intensity images for four different metasurface sub-arrays under the LCP and RCP light.

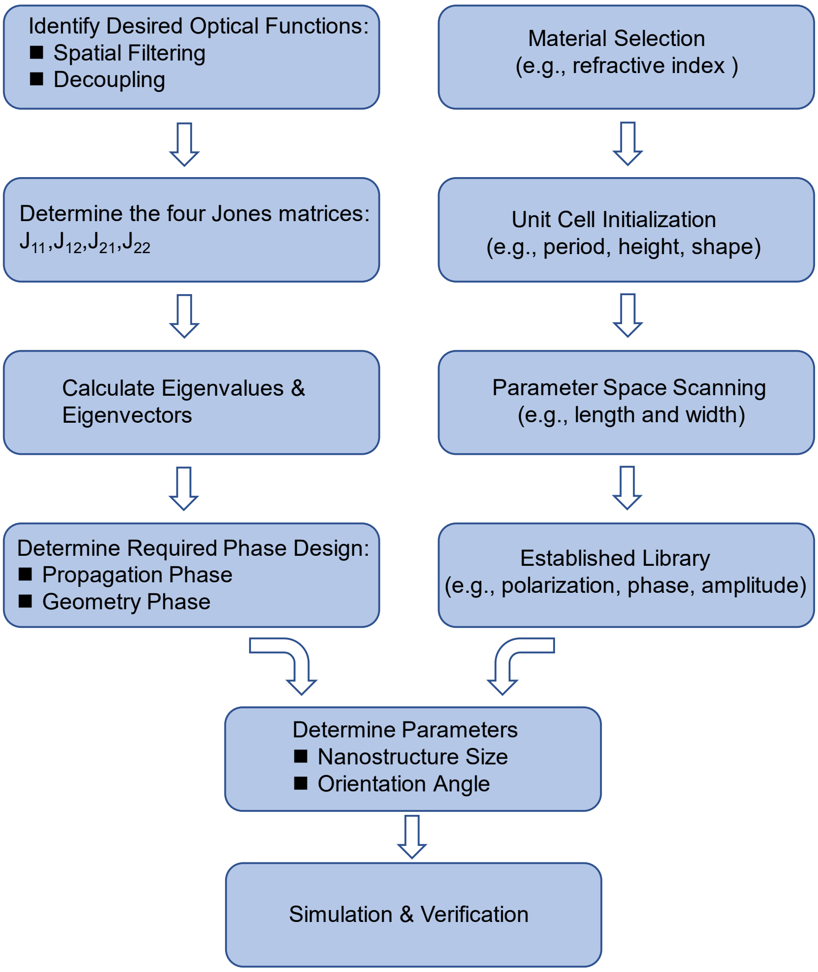

Figure S3 | Metasurface Design Process. The process begins with a functional requirements analysis to clarify the desired optical functions, such as spatial filtering and information decoupling, to visualize a fully two-dimensional transparent flow field. Based on these functional requirements, the Jones matrix of each metasurface sub-array is determined. Matrix calculations are then performed to solve for the eigenvalues and eigenvectors of the Jones matrix, which define the propagation and geometric phases required at each spatial location (x, y). In the materials and unit cell structure design, the working wavelength is considered to select suitable materials and determine initial design parameters, such as the shape, size, and orientation of the unit structures. Subsequently, by scanning the parameter space, the relationships between polarization, phase, amplitude, and structural parameters are established. Using the required propagation and geometric phases along with the constructed library, the specific size and orientation of the nanostructure at each spatial location are determined. Finally, finite-difference time-domain (FDTD) simulations are conducted to evaluate whether the metasurface design meets the specified optical functionality.

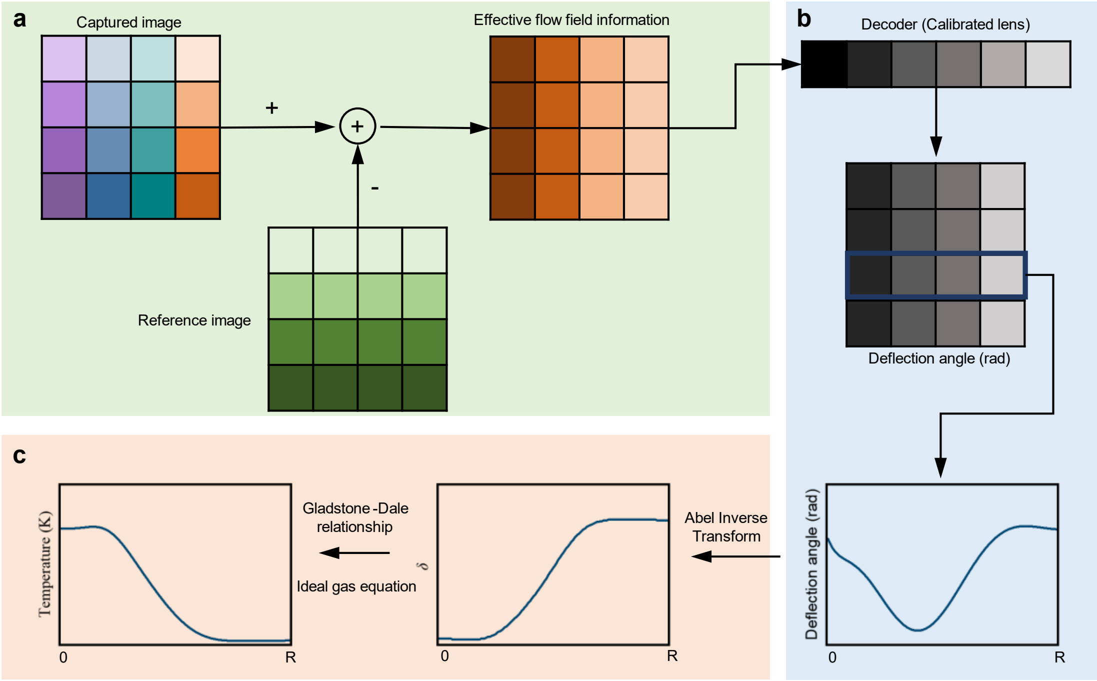

Figure S4 | Flowchart of the reconstruction algorithm. (**a**) Captured flow field images are combined with reference images taken in the absence of a flow field to obtain effective flow field information. (**b**) The distribution of light deflection angles induced by the flow field is calculated using the standard photometry method. The visualization images of the calibration lens and the flow field are obtained under the same system parameters. (**c**) Based on the assumption of axial symmetry, the refractive index distribution within the flow field can be obtained using the Abel inversion transform. Further analysis using the Gladstone-Dale relationship and the ideal gas equation yields the density information and temperature distribution of the flow field. R represents the distance from the axis of symmetry, and δ represents the refractive index distribution.

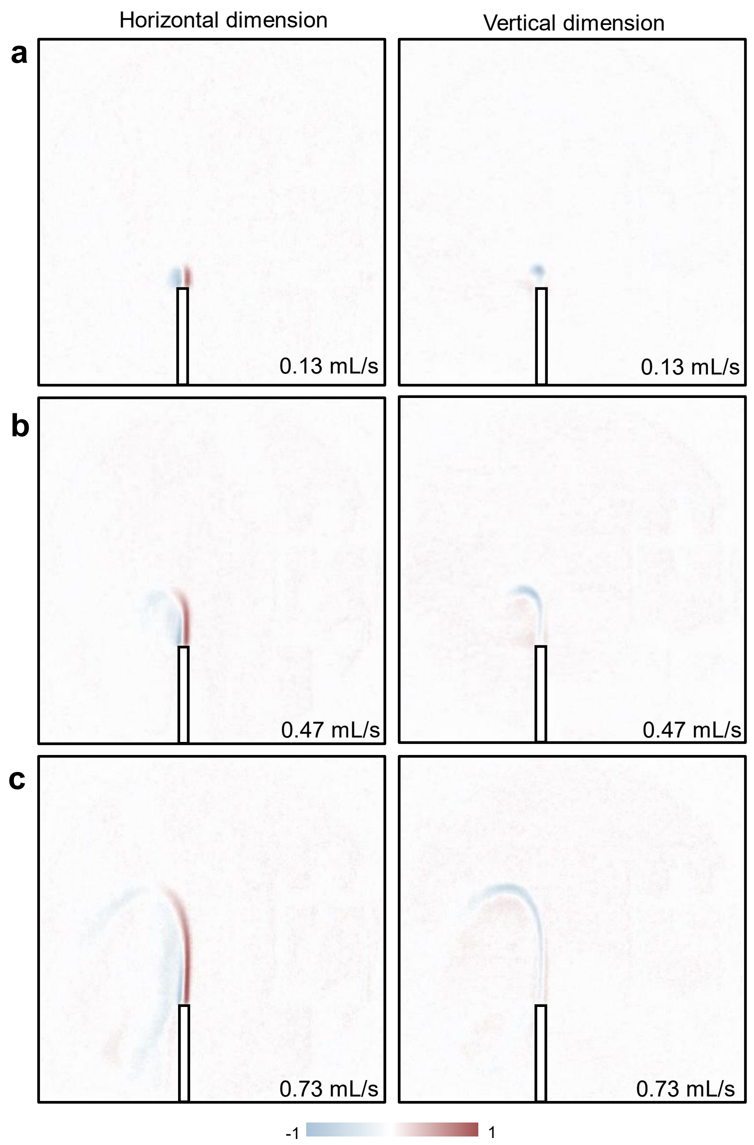

Figure S5 | Detection of minor gas leaks. Flow field images of carbon dioxide gas at flow rates of 0.13 mL·s^{-1} (**a**), 0.47 mL·s^{-1} (**b**), and 0.73 mL·s^{-1} (**c**), respectively. The black solid line in the figure represents the carbon dioxide gas outlet.

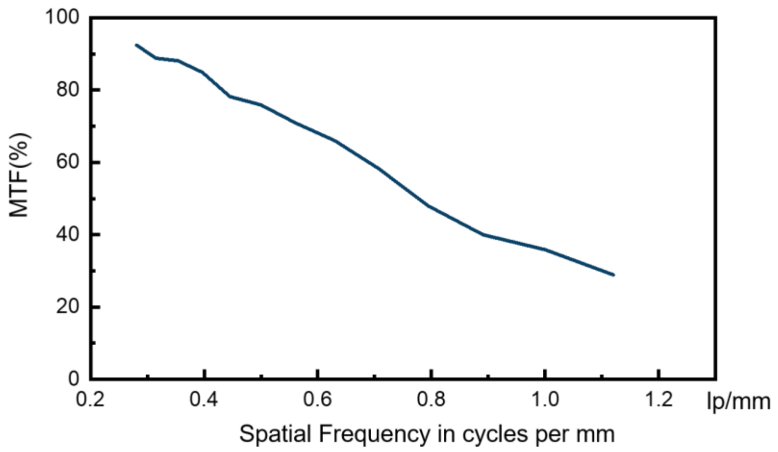

Figure S6 | MTF curve of the system.

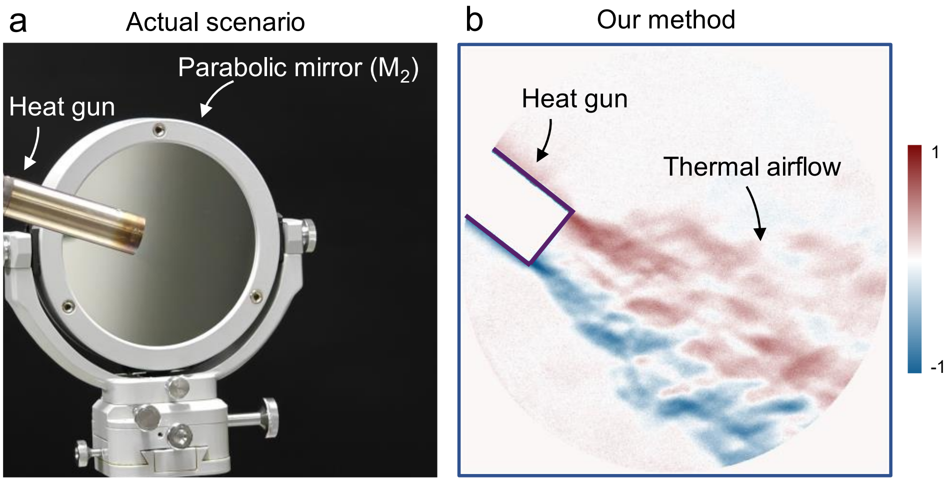

Figure S7 | An example illustrating the actual scenario in the experiment. (a) Actual scene of the heat gun experiment. (b) Flow field image (vertical dimension) obtained by our method. The dark purple solid line represents the position of the heat gun.

References:

1. A. Arbabi, Y. Horie, M. Bagheri, A. Faraon, Dielectric metasurfaces for complete control of phase and polarization with subwavelength spatial resolution and high transmission. *Nat. Nanotechnol.* **10**, 937-943(2015).
2. J. P. B. Mueller, N. A. Rubin, R. C. Devlin, B. Groever, F. Capasso, Metasurface Polarization Optics: Independent Phase Control of Arbitrary Orthogonal States of Polarization. *Phys. Rev. Lett*. **118**, 113901 (2017).
3. Q. Fan, M. Liu, C. Zhang, W. Zhu, Y. Wang, P. Lin, F. Yan, L. Chen, H. J. Lezec, Y. Lu, A. Agrawal, T. Xu, Independent amplitude control of arbitrary orthogonal states of polarization via dielectric metasurfaces. *Phys. Rev. Lett*. **125**, 267402(2020).
4. M. V. Berry, The adiabatic phase and Pancharatnam's phase for polarized light. *J. Mod. Opt.* **34**, 1401-1407 (1987).
5. Z. E. Bomzon, G. Biener, V. Kleiner, E. Hasman, Space-variant Pancharatnam–Berry phase optical elements with computer-generated subwavelength gratings. *Opt. Lett.* **27**, 1141-1143(2002).
6. G. Pretzier, A new method for numerical Abel-inversion. *Z. Naturforsch. A* **46**, 639-641(1991).
